# Efficacy of fennel (*Foeniculum vulgare*) and anise (*Pimpinella anisum*) essential oils as anaesthesics in common carp (*Cyprinus carpio* L. 1758)

**DOI:** 10.1007/s10695-024-01341-6

**Published:** 2024-04-17

**Authors:** Secil Metin, Hakan Didinen, Nalan Ozgur Yigit, Hasan Eralp, Ozlem Ozmen, Meric Lutfi Avsever

**Affiliations:** 1 Egirdir Fisheries Faculty, Isparta University of Applied Sciences, Isparta, Turkey; 2 Yalvaç Vocational School of Technical Sciences, Isparta University of Applied Sciences, Isparta, Turkey; 3 Department of Pathology, Faculty of Veterinary Medicine, Burdur Mehmet Akif Ersoy University, Burdur, Turkey; 4 Akhisar Vocation of High School, Plant and Animal Production Department, Manisa Celal Bayar University, Manisa, Turkey

**Keywords:** Natural anaesthesic, Essential oil, Carp, Histopathology, Blood parameters

## Abstract

In this study, the anaesthetic effects of fennel and anise essential oils were investigated on common carp. Fish (10 ± 0.45 g) were exposed to nine concentrations of essential oils (5, 10, 20, 50, 100, 200, 300, 400 and 500 mg L^−1^). Additionally, the histopathological effects on the fish tissues including gill, skin and hepatopancreas and physiological effects on some blood parameters (Na^+^, K^+^, Ca^+2^, Cl^−^, total plasma protein and glucose) of essential oils were investigated in carp. At the end of the experiment, fennel oil showed an anaesthetic effect at a concentration of 500 mg L^−1^ in carp (anaesthesia induction and recovery times were 308 and 472 s, respectively). Anise essential oil showed deep anaesthesia at a concentration of 100 mg L^−1^, but anaesthesia induction time was found to be very long (20 min). In addition, anise oil at concentrations above 100 mg L^−1^ caused 10% mortality in fish. Blood parameters except glucose level in both essential oils were unchanged during deep anaesthesia in carp. However, plasma glucose levels were found lower in fish anaesthetized with anise oil than control and fennel groups (*P < 0.05*). At the histopathological examination, no pathological findings were observed in any organ of fish in the fennel group. However, severe hyperemia and inflammatory cell infiltrations in gills, erosive lesions in the skin and slight inflammatory reactions in the skin were observed in the anise group. The present study demonstrated that fennel essential oil at 500 mg L^−1^ concentration can be used as an effective and safe anaesthetic in common carp, but anise essential oil is not suitable.

## Introduction

Anaesthesia is mostly utilized in aquaculture, which decreases fish stress and prevents physical injury during fish handling through routine practices (Inoue et al. 2003; Altun and Danabas 2006). Many chemical anaesthetics have been used in aquaculture until now (Altun and Danabas 2006; Priborsky and Velisek 2018). However, they have undesirable side effects (Palić et al. 2006). In the selection criteria of anaesthetics, induction and recovery time of anaesthesia are important (Martins et al. 2019). Anaesthetics should be effective in low concentrations and have no toxic effect on fish (Seyidoglu and Yagcılar 2020). Therefore, the search for natural anaesthetic agents that are reliable and effective in fish has increased in recent years. Previous studies have investigated effects of plant essential oils as effective, safe and inexpensive new anaesthetics (Inoue et al. 2003; Velisek et al. 2005; Seyidoglu and Yagcılar 2020).

Fennel and anise are important members of the Apiaceae (Umbelliferae) family (Telci et al. 2009; Sun et al. 2019). Both essential oils have antibacterial, antifungal, anti-inflammatory, analgesic, antioxidant and sedative effects (Kooti et al. 2015; Sun et al. 2019). The main component of fennel and anise is anethole (Telci et al. 2009; Orav et al. 2008). Domiciano et al. (2013) reported that anethole observed a sedative and anaesthetic effect. There is a study on the anaesthetic effectiveness of fennel and anise essential oils in fish. Ugur (2019) reported that fennel essential oil showed an anaesthetic effect on rainbow trout. Anise essential oil has been demonstrated anaesthetic effect on zebrafish (Seyidoglu and Yagcılar 2020). In previous studies, different plant essential oils and active components have been studied as anaesthetic in common carp (Roohi and Imanpoor 2015; Mirghaed et al. 2016; Mazandarani and Hoseini 2017; Mazandarani et al. 2017; Rakhshani et al. 2018; Khumpirapang et al. 2018; Yousefi et al. 2018a, 2018b; Al-Niaem et al. 2019; Krasteva et al. 2021; Metin et al. 2022).

The present study investigated the anaesthetic effects of fennel (*Foeniculum vulgare*) and anise (*Pimpinella anisum*) essential oils as a new anaesthetic agent for common carp (*Cyprinus carpio*) and their effect on the histopathology and some blood parameters for the first time.

## Materials and methods

### Plant materials and essential oil isolation

In this study, fennel and anise were used as plant materials. The plants were taken from the Isparta region and were dried in the shadow and the stem parts were separated after drying. The essential oil was isolated with the distillation process using a Clevenger apparatus in the Industrial Crops Laboratory in the Faculty of Agriculture, Isparta Applied Sciences University. Five hundred grams of plant samples in 1.5 L water were extracted by hydro-distilllation for 3 h using the Clevenger apparatus according to the standard procedure described in European Pharmacopoeia for determining the oil content (v/w %).

### Essential oil analyses

The composition of essential oil was analyzed using gas chromatography–mass spectrometry (GC-MS). Each component was identified by comparison from the Wiley, Nist, Tutor and FFNSC Library of Mass Spectra. The component amount was determined by proportioning the relative blocks of the peak areas to the total peak area.

### Experimental design

The experiment was carried out at the Aquarium Units of Egirdir Fisheries Faculty, Isparta Applied Sciences University, Turkey. Common carp (10 ± 0.45 g) were obtained from the Mediterranean Fisheries Research, Production and Training Institute, Antalya, Turkey. One hundred twenty carp were placed in 2 adaptation tanks (450 L) with aeration. Fish were acclimated for 15 days before the beginning of the experiments. Fish were fed by hand, ad libitum twice daily with commercial feed. The tanks were cleaned from residual feed and faeces by siphoning out. Water temperature and dissolved oxygen were measured as 28 °C and 6 mg L^−1^. After 15 days, the anaesthetic effects of fennel and anise essential oils at 5, 10, 20, 50, 100, 200, 300, 400 and 500 mg L^−1^ concentrations were investigated in common carp. Induction and recovery stages were evaluated according to Keene et al. (1998) (Table 1). Ten fish were used to determine anaesthesia induction and recovery time. The fish were caught with hand nets from the holding tanks and placed individually into the aquariums (10 L) containing anaesthetic solution with continuous aeration. The duration of each stage was recorded with a stopwatch. The time to reach stage 4 anaesthesia was recorded and then the fish was caught and placed into the recovery aquarium containing clean water to record recovery time. After recovery, abnormal behaviour (swimming, position in the water column etc.) and mortalities were observed for 30 min (Can et al. 2019).

**Table 1:** Anesthesia stages in fish

Stage	Description	Behaviour exhibited
1	Light sedation	Equilibrium normal, slow swimming, decreased reactivity to external stimuli, slight decrease in opercular rate
2	Deep sedation	Equilibrium normal, voluntary swimming still possible, slight decrease in opercular rate no response to weak external stimulus
3	Light anaesthesia	Partial loss of equilibrium, swimming erratic, increased opercular rate, reactive only to strong tactile and vibrational stimuli
4	Deep anaesthesia	Total loss equilibrium, lying on one side without movement, opercular movements slow and irregular; loss of all reflexes
	Recovery	Regaining equilibrium and active swimming

### Histopathological examination

Tissue samples (gill, skin and hepatopancreas) were obtained from fish treated with 500 mg L^−1^ concentration of both essential oils and control fish. Five fish from each group were euthanized by spinal cord section for histopathological samples. Samples were fixed in 10% neutral formalin and processed. The samples were embedded in paraffin, and 5 μm sections were taken by microtome and the sections were stained with hematoxylin and eosin.

### Blood analyses

Blood samples from fish were taken immediately after deep anaesthesia at 500 mg L^−1^ concentration of both essential oils and control fish. The blood was collected from the caudal vein and was placed in non-heparinized tubes and, after clotting, centrifuged at 3000 g for 5 min. Plasma analyses (glucose, total plasma protein, chloride, total calcium, sodium, potassium) were performed on an auto-analyzer (Abbott ARCHITECT ci8200).

### Statistical analysis

The homogeneity of variance and normality for data (anaesthesia induction and recovery times) were checked using Levene’s test and the Shaphiro-Wilk test, respectively. Then, data were assessed by one-way analysis of variance ANOVA SPSS 16.0 package program (SPSS Inc., Chicago, IL, USA). Duncan test was used to determine the significant variation (*p* < 0.05). Regression equations were used to explain the relationship between anaesthetic concentrations and induction/recovery times.

## Results

### Chemical composition of essential oils

The components of fennel and anise essential oils by GC-MS are given in Tables 2 and 3. A total of 28 components in fennel and 18 in anise were identified. Anethole (75.49%), limonene (5.96%), p-allylanisole (5.94%), fenchone (3.70%) and anisaldehyde (3.19%) in fennel and anethole (88.01%), p-allylanisole (3.27%) and anisaldehyde (3.82%) in anise were determined as main components (Tables 2 and 3).

**Table 2:** Essential oil components of fennel (%)

Component	Rt	Content %
Anethole	26.021	75.49
Limonene	10.311	5.96
p-Allylanisole	19.896	5.94
Fenchone	13.301	3.70
Anisaldehyde	23.497	3.19
Benzaldehyde, 4-(1-methylethyl)-	22.616	0.85
Cymol	10.066	0.76
Gamma terpinene	11.718	0.63
Alpha pınene	6.493	0.58
Eucalyptol (1,8-cıneole)	10.437	0.49
2-Cyclohexen-1-one, 2-methyl-5-(1-methylethenyl)-, (R)-	22.755	0.49
Acetylphenylcarbinol	26.089	0.32
Cis-ocimene	10.612	0.32
Camphene	7.874	0.24
1-Methoxy-4-(oxiran-2-yl) methyl benzene	31.558	0.22
Acetonylanisole <para->	31.717	0.19
Beta myrcene	8.509	0.13
l-.beta.-pinene	8.074	0.10
Camphor	16.597	0.08
Cis-p-mentha-2,8-dien-1-ol	16.081	0.07
Linalool	13.988	0.06
l-Phellandrene	9.236	0.05
4-Terpineol	18.721	0.04
Beta fenchyl alcohol	19.665	0.03
Beta ocımene Y	11.126	0.02
Beta phellandrene	7.063	0.02
Cıs-lımonene oxıde	15.817	0.01
Alpha-terpınolene	13.110	0.01

**Table 3:** Essential oil components of anise (%)

Component	Rt	Content %
Anethole	26.014	88.01
Anisaldehyde	23.486	3.82
p-Allylanisole	19.865	3.27
Humulen	37.690	1.17
Benzaldehyde, 4-(1-methylethyl)-	22.615	1.16
Cymol	10.065	0.84
Gamma terpinene	11.722	0.66
Limonene	10.292	0.31
2-(1-E-propenyl)-4-methoxyphenyl 2-methylbutanoate	58.249	0.27
Para acetonylanisole	31.729	0.10
Alpha himachalene	35.840	0.09
Ar-curcumene	38.049	0.08
l-beta pinene	8.079	0.06
Linalool	13.996	0.05
Myrcene	8.510	0.03
Cis-ocimene	10.611	0.03
l-phellandrene	9.245	0.03
Beta bisabolene	39.751	0.02

### Anaesthetic efficacy of fennel and anise essential oil

**Fig. 1:**
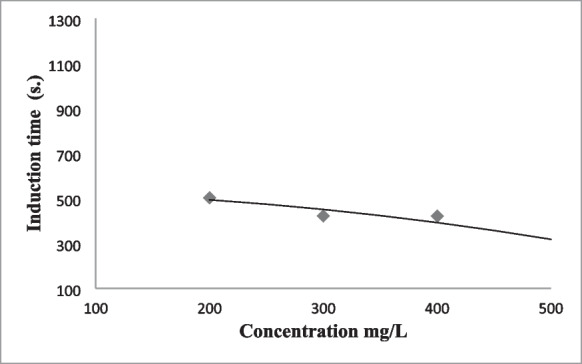
Relationship between fennel essential oil concentration with anesthesia induction time of common carp (Stage 4)

**Fig. 2:**
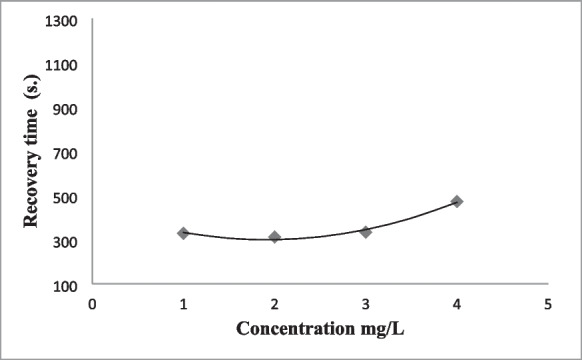
Relationship between fennel essential oil concentration with recovery time of common carp

The anaesthesia induction time of the fish anaesthetized with fennel essential oil decreased with the increase in concentration (*P < 0.05*). Recovery time of fish anaesthetized with fennel increased with increasing concentration (*P < 0.05*). Fennel oil concentrations between 50 and 500 mg L^−1^ provided deep sedation (Stage 2) in carp (Table 4). 100–500 mg L^−1^ concentrations of fennel essential oil showed deep anaesthesia (Stage 4) in fish. Considering the ideal anaesthesia times, fennel oil at 500 mg L^−1^ concentration was found suitable for deep anaesthesia in fish. Anaesthesia induction and recovery times for 500 mg L^−1^ fennel oil were 308 and 472 s, respectively (Table 4). Mortality or abnormal behaviour in fish was not recorded after anaesthesia with fennel essential oil. There was a relationship between the anaesthesia induction (Stage 4) and recovery times with fennel essential oil concentrations according to regression analysis (coefficient values: 0.90 for anaesthesia induction time, 0.98 for recovery time) (Figs. 1 and 2).

**Table 4:** Anesthetic effect of fennel oil on carp fish

Dose (mg L^−1^)	Induction time(s)				Recovery time(s)
	Anaesthesia level				
	I	II	III	IV	
5		-	-	-	
10	534.00 ± 12.00^a^	-	-	-	
20	262.50 ± 7.50^b^	-	-	-	
50	221.00 ± 14.00^c^	595.50 ± 9.50^a^	1126.50 ± 13.50^a^	-	
100	199.50 ± 10.50^cd^	352.50 ± 11.50^b^	554.50 ± 7.50^b^	-	
200	172.00 ± 8.00^de^	342.50 ± 10.50^b^	372.50 ± 7.50^c^	503.00 ± 7.00^a^	312.00 ± 8.00^b^
300	144.50 ± 10.50^ef^	247.50 ± 9.50^c^	351.00 ± 9.00^c^	421.50 ± 13.50^b^	328.00 ± 12.00^b^
400	133.50 ± 10.50^f^	221.50 ± 8.50^cd^	290.50 ± 9.50^d^	421.00 ± 9.00^b^	333.50 ± 6.50^b^
500	116.00 ± 7.00^f^	203.50 ± 8.50^d^	254.00 ± 11.00^e^	308.00 ± 7.00^c^	472.50 ± 7.50^a^
Equation*	*y* = 872.34*x*^−0.32^	*y* = 3102*x*^−0.439^	*y* = 10,417*x*^−0.604^	*y* = −0.0008*x*^2^ − 0.0342*x* + 531.68	*y* = 38.75*x*^2^ − 148.25*x* + 441.5
*R*^2^	0.91	0.94	0.95	0.90	0.98

Data are presented as mean ± SD (*n* = 10). Values superscript with different letters at same column are significantly different (*p* < 0.05).

- No anesthetic effect

*Relationships between concentration × anesthesia induction or recovery time in common carp, exposed to the fennel essential oil.

Fish anaesthetized with anise essential oil reached sedation at all concentrations. Anise essential oil showed deep anaesthesia (Stage 4) at a concentration of 100 mg L^−1^. However, anaesthesia induction at this concentration was found very long (20 min) in considering ideal anaesthesia times. In addition, anise essential oil caused 10% mortality in fish, although it showed an anaesthetic effect at concentrations of 200–500 mg L^−1^ (Table 5). Therefore, it was found to be unsuitable for anaesthesia in common carp.

**Table 5:** Anesthetic effect of anise oil on carp fish

Dose (mg L^−1^)	Induction time(s)				Recovery time(s)
	Anaesthesia level				
	I	II	III	IV	
5	750.00 ± 30.00^a^	-	-	-	
10	462.50 ± 17.50^b^	-	-	-	
20	323.50 ± 18.50^c^	1420.00 ± 20.00^a^	-	-	
50	245.00 ± 35.00^d^	523.50 ± 16.50^b^	823.50 ± 16.50^a^	-	
100	219.00 ± 21.00^d^	263.00 ± 7.00^c^	489.00 ± 6.00^b^	1306.50 ± 13.50^a^	2194 ± 26.87 ^a^
200	110.00 ± 10.00^e^	246.50 ± 11.50^c^	354.50 ± 5.50^c^	1301.00 ± 19.00^a^	1800 ± 57.92 ^b^
300	106.50 ± 13.50^e^	227.00 ± 13.00^c^	349.00 ± 11.00^c^	1252.50 ± 7.50^b^	864.50 ± 5.50^c^
400	-	216.50 ± 13.50^c^	247.50 ± 7.50^d^	361.00 ± 9.00^c^	388.00 ± 12.00 ^d^
500	-	169.00 ± 11.00^d^	205.50 ± 14.50^e^	311.00 ± 4.00^d^	395.50 ± 34.50^d^
Equation*	*y* = 1432.6*x*^−0.457^	*y* = 6002.8*x*^−0.587^	*y* = 6825.3*x*^−0.552^	*y* = −0.0067*x*^2^ + 1.0633*x* + 1319.7	*y* = 0.009*x*^2^ – 10.418*x* + 3262.1
*R*^2^	0.96	0.88	0.96	0.84	0.95

Data are presented as mean ± SD (*n* = 10). Values superscript with different letters at same column are significantly different (*p* < 0.05).

- No anesthetic effect

*Relationships between concentration × anesthesia induction or recovery time in common carp, exposed to the anise essential oil.

### Blood parameters

No differences were observed for Ca, Cl, K, Na and total plasma protein after anaesthesia with essential oils (Table 6) (*P > 0.05*). Plasma glucose levels in carp were found to be similar in the fennel essential group to the control group (*P > 0.05*). Glucose levels decreased in fish anaesthetized with anise essential oil (*P < 0.05*).

**Table 6:** Some blood parameters of carp

Groups	Ca, mg dL^−1^	Cl, mmol L^−1^	K, mEq L^−1^	Na, mEq L^−1^	Glucose, mg dL^−1^	Total protein, g dL^−1^
Anise 500 mg L^−1^	9.05 ± 0.05	110.50 ± 0.50	2.70 ± 1.10	129.50 ± 0.50	82.50 ± 5.50^a^	2.25 ± 0.15
Fennel 500 mg L^−1^	9.30 ± 0.10	111.00 ± 1.00	2.40 ± 0.20	131.50 ± 1.50	101.50 ± 0.50^b^	2.25 ± 0.05
Control	9.55 ± 0.35	112.50 ± 0.50	2.70 ± 0.10	133.50 ± 0.50	98.50 ± 3.50^b^	2.15 ± 0.15

Data are presented as mean ± SEM. Values superscript with different letters at same column are significantly (*p* < 0.05) different

### Histopathological examination

**Fig. 3:**
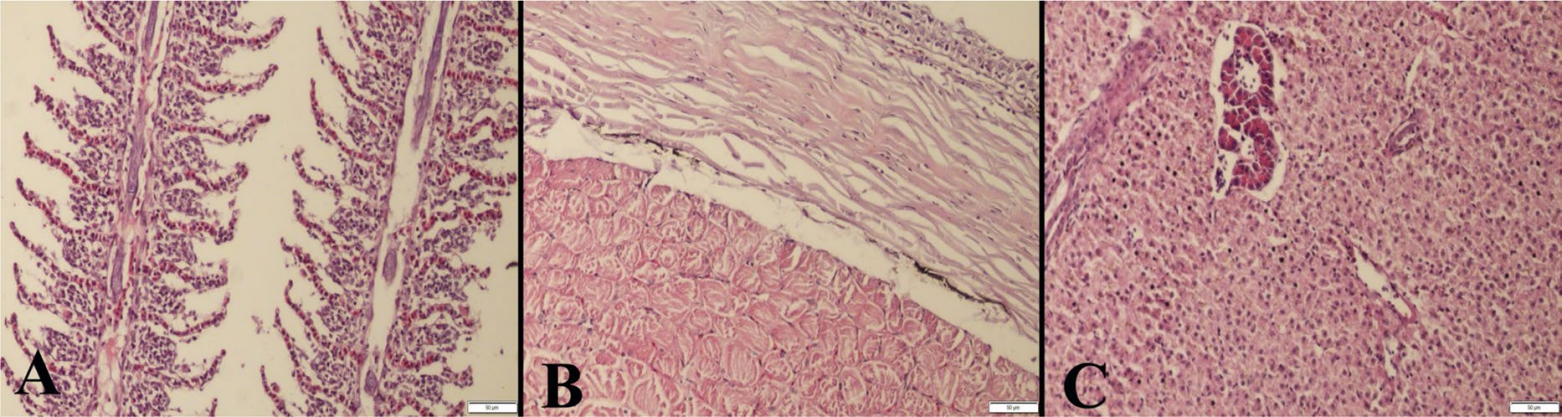
Representative histological appearance of the fennel group (500 mg L^−1^), normal **A** gill, **B** skin and **C** hepatopancreas architecture, HE, bars = 50μm.

**Fig. 4:**
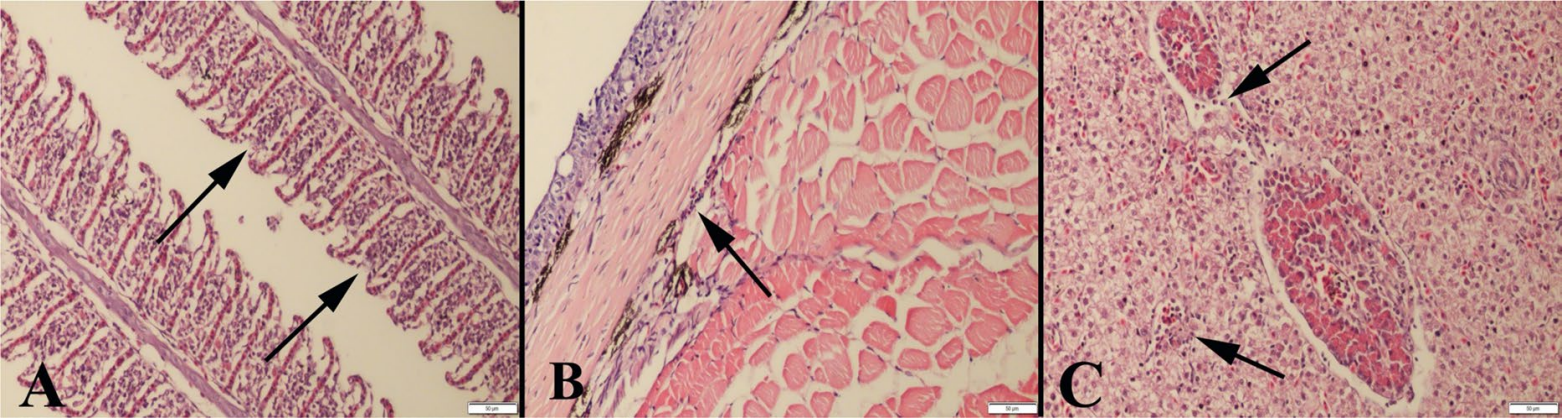
Microscopical appearance of the anise group (500 mg L^−1^), **A** marked inflammatory reaction in gill (arrows), **B** slight inflammation in skin (arrow) and **C** slight inflammatory cell infiltrations (arrows) in hepatopancreas, HE, bars = 50μm

In this study, skin, hepatopancreases and gills of the fennel group displayed normal histology (Fig. 3). In the anise group, the gills showed marked hyperemia and intense inflammatory cell infiltrations. Additionally, erosive lesions in some parts of the skin and mild inflammatory reactions in muscles were observed. In the hepatopancreases of three fish, there were slight inflammatory cell infiltrations (Fig. 4).

## Discussion

Herbal essential oils have been evaluated as new anaesthetics agents in fish. The anaesthetic efficacy of essential oil is related to its chemical composition. Anethole, the main component of fennel and anise essential oils, has been reported to have anaesthetic properties (Figueredo et al. 2020; Senatore et al. 2013; Domiciano et al. 2013). In the present study, anethole was also found as the main component of fennel (75.49%) and anise (88.01%) essential oils. Similarly, Figueredo et al. (2020) reported a high rate of anethole in anise essential oil. Senatore et al. (2013) also noted high anethole concentration in fennel.

It is critical to determine the effective anaesthetic concentration in each fish species (Aydın and Barbas 2020). The ideal anaesthetic effect allows the fish to enter anaesthesia in less than 6 min and recover in less than 10 min (King et al. 2005). According to this criterion, the lowest effective concentration of fennel essential oil for deep anaesthesia was determined as 500 mg L^−1^ in the present study. It was found that the anaesthesia induction and recovery times at this concentration for carp were 308 and 472 s., respectively. Similarly, Ugur (2019) also noted that the anaesthetic effect of fennel (*F. vulgare*) essential oil was obtained at 400 mg L^–1^ (188 s induction and 136.5 s recovery time) in rainbow trout.

In this study, anise essential oil showed deep anaesthesia (Stage 4) at a concentration of 100 mg L^−1^ in common carp. However, anaesthesia induction at this concentration was found very long (20 min) in considering ideal anaesthesia times. In addition, concentrations above 100 mg L^−1^ anise oil caused 10% mortality in fish. For this reason, it has been observed that anise is not suitable for anaesthesia in carp. Similarly, Seyidoglu and Yagcılar (2020) reported that anise at concentrations of 0.1–30 mg L^−1^ did not show an anaesthetic effect and caused mortality in zebrafish.

In this study, anise and fennel were found to have similar essential oil content (especially high amounts of anethole). Anise oil caused mortality in fish, but no mortality was observed with fennel oil. This can be explained by the effect of toxic metabolites, even in low amounts, found in anise oil. It is also possible to modulate the activity of the main components by other small molecules (Bakkali et al. 2008).

Electrolyte balance is one of the important indicators in determining the suitability of the anaesthesia process in fish (Honorato et al. 2014). In this study, Na^+^, K^+^, Ca^+2^ and Cl^−^ did not change in deep anaesthesia with fennel and anise essential oils in carp. Similarly, Souza et al. (2017) reported that blood ions (Na^+^, K^+^, Ca^+2^) were not affected during deep anaesthesia with *Lippia alba* essential oil in catfish. Honorato et al. (2014) found similar results in the blood of Amazonian catfish (*Leiarius marmoratus*) anaesthetized with eugenol.

Glucose level is an important indicator in determining the secondary effects of stress (Maricchiolo and Genovese 2011; Pankhurst 2011; Silva et al. 2015). Fish exposed to stress increase energy metabolism to cope with stress response, and glucose is used as the main energy fuel (Wendelaar Bonga 1997; Lupatsch et al. 2010; Jiang et al. 2017). Stress causes increases in glucose levels in fish (Malini et al. 2018). In the present study, plasma glucose levels after anaesthesia were not affected in the fennel group. Thus, no stress-related increase in plasma glucose was observed following exposure of carp to fennel essential oils. Similar results were obtained in Nile tilapia anaesthetized with *Lippia alba* (Hohlenwerger et al. 2016) and *Aloysia triphylla* (Teixeira et al. 2018) and *Ocimum basilicum* essential oils (Ventura et al. 2020). In the present study, plasma glucose levels after anaesthesia decreased in the anise group. Similarly, Roohi and Imanpoor (2015) noted that in spearmint oil at 5 mL L^−1^ doses in carp, the glucose level determined after recovery was found to be lower than during anaesthesia. On the other hand, Velisek et al. (2005) reported that glucose levels increased in carp immediately after anaesthesia with clove oil. Differences in results in glucose response may be due to different essential oil, dosage, exposure time, fish species and temperature.

Fish in stressful situations may experience altered plasma protein values due to the synthesis of the hormone cortisol (Cunha et al. 2000). In this study, total plasma protein did not change in anaesthesia with fennel and anise essential oils in carp. Similarly, the use of *Hesperozygis ringens* essential oil for silver catfish (*Rhamdia quelen*) anaesthesia did not change plasma protein values (Toni et al. 2014).

There is no study on the histopathological effects of using anise and fennel essential oil as anaesthetics in fish. In the present study, no pathological findings were found in the skin, gill and hepatopancreas of fish anaesthetized with fennel essential oils. Similar results were reported for clove oil in common carp (Velisek et al. 2005) and for lavender in silver carp (Golshan et al. 2018). In the present study, anise essential oil caused severe hyperemia and inflammatory cell infiltrations in gills, erosive lesions in the skin, slight inflammatory reaction in muscle and hepatopancreases in carp. Similarly, Yigit and Kocaayan (2023) reported that thyme essential oil caused marked hyperemia, oedema, inflammatory cell infiltrations and desquamation in gills. de Lima et al. (2021) also reported that eugenol caused moderate to severe histological changes in the gills of the Amazonian freshwater stingray, *Potamotrygon wallacei*. Brandão et al. (2021) reported hypertrophy and hyperplasia of the lamellar epithelium, lamellar fusion and proliferation of chloride cells in *Colossoma macropomum* anaesthetized with essential oils of *Aloysia triphylla*, *Lippia sidoides* and *Mentha piperita*. These effects are thought to result from the accumulation of anise essential oil in the gills for a long time and in high concentrations, leading to harmful effects.

As a result, in this study, the anaesthetic effects of fennel and anise essential oils and their effect on histopathology and some blood parameters were investigated in common carp for the first time. This study demonstrated that fennel essential oil at 500 mg L^−1^ is an ideal concentration for the anaesthesic effect in common carp. No microscopical lesions of fennel essential oil were found in fish. Anise essential oil showed anaesthetic effects at concentrations of 100–500 mg L^−1^, but caused mortality in fish. In addition, anise caused histopathological findings in gills, hepatopancreas and skin. As a result of this study, fennel essential oil at 500 mg L^−1^ concentration can be used as a natural anaesthetic in carp culture. However, anise was found to be unsuitable for anaesthesia in common carp. Future studies need to evaluate the effect of fennel essential oil on other blood parameters (cortisol, hematological responses) in fish and its effectiveness as an anaesthetic in different fish species.

## Data Availability

No datasets were generated or analysed during the current study.
